# Comparative transcriptomic analysis reveals female-biased olfactory genes potentially involved in plant volatile-mediated oviposition behavior of *Bactrocera dorsalis*

**DOI:** 10.1186/s12864-020-07325-z

**Published:** 2021-01-06

**Authors:** Li Xu, Kai-Yue Tang, Xiao-Feng Chen, Yong Tao, Hong-Bo Jiang, Jin-Jun Wang

**Affiliations:** 1 Key Laboratory of Entomology and Pest Control Engineering, College of Plant Protection, Southwest University, Chongqing, 400715 China; 2 State Cultivation Base of Crop Stress Biology for Southern Mountainous Land, Academy of Agricultural Sciences, Southwest University, Chongqing, 400715 China

**Keywords:** *Bactrocera dorsalis*, Antenna, Chemosensory, Benzothiazole, 1-octen-3-ol

## Abstract

**Background:**

Olfactory systems take on important tasks to distinguish salient information from a complex olfactory environment, such as locating hosts, mating, aggression, selecting oviposition sites, and avoiding predators. The olfactory system of an adult insect consists of two pairs of main olfactory appendages on the head, the antennae, and the palps, which are covered with sensilla. Benzothiazole and 1-octen-3-ol could elicit oviposition behavior in gravid *B. dorsalis* are regarded as oviposition stimulants. However, the mechanism for how *B. dorsalis* percepts benzothiazole and 1-octen-3-ol still remains unknown.

**Results:**

We conducted a comparative analysis of the antennal transcriptomes in different genders of *B. dorsalis* using Illumina RNA sequencing (RNA-seq). We identified a total of 1571 differentially expressed genes (DEGs) among the two sexes, including 450 female-biased genes and 1121 male-biased genes. Among these DEGs, we screened out 24 olfaction-related genes and validated them by qRT-PCR. The expression patterns of these genes in different body parts were further determined. In addition, we detected the expression profiles of the screened female-biased chemosensory genes in virgin and mated female flies. Furthermore, the oviposition stimulants-induced expression profilings were used to identify chemosensory genes potentially responsible for benzothiazole and 1-octen-3-ol perception in this fly.

**Conclusions:**

The results from this study provided fundamental data of chemosensory DEGs in the *B. dorsalis* antenna. The odorant exposure assays we employed lay a solid foundation for the further research regarding the molecular mechanism of benzothiazole and 1-octen-3-ol mediated oviposition behavior in *B. dorsalis*.

**Supplementary Information:**

The online version contains supplementary material available at 10.1186/s12864-020-07325-z.

## Background

The oriental fruit fly, *Bactrocera dorsalis* (Hendel), is a destructive agricultural pest that is widely distributed in many areas of the Asian countries [1]. It causes severe economic loss and trade restrictions to vegetables and fruits by ovipositing inside more than 450 host plant species (USDA, 2016). Recently, owing to its strong environmental adaptiveness, invasive ability [2], and the quick development of resistance [3], the damage produced by this pest has become increasingly serious. Therefore, reducing damage by *B. dorsalis* is becoming a focus of research efforts worldwide. Currently, the most economical and effective way to control *B. dorsalis* is olfaction-based trapping [4], which indicates that the olfaction system plays an important role in management of this pest.

Olfactory systems take on important tasks to distinguish salient information from a complex olfactory environment [5]. It is reported that the exceptional sensing ability plays essential roles in locating hosts, mating, aggression, selecting oviposition sites, choosing habitats, and avoiding predators [6]. The olfactory system of an adult insect consists of two pairs of main olfactory appendages on the head, the antennae, and the palps [7], which are covered with sensilla. The peripheral olfactory sensing of insects mainly relies on the olfactory sensory neurons (OSNs), which are located at the olfactory sensilla of the antenna. Recently, RNA sequencing and bioinformatics-based approaches have been rapidly developed to analyze insect communication systems [8]. So far, many antennal transcriptome projects from various insect species have been completed, such as *Helicoverpa armigera* [9], *Chilo suppressalis* [10], *Cotesia vestalis* [11] and *Ostrinia furnacalis* [12]. It has demonstrated that chemoreception involves several families of proteins including odorant binding proteins (OBPs), sensory neuron membrane proteins (SNMPs), chemosensory proteins (CSPs), odorant degrading enzymes (ODEs), odorant receptors (ORs), gustatory receptors (GRs), and ionotropic receptors (IRs) [6].

Phytochemicals from host plants are known to affect sexual behaviors and physiology in a wide range of insects. In several phytophagous insect species, males are attracted to plant volatile compounds to increase the possibility of encountering females [13]. In *B. dorsalis*, it is well known that methyl eugenol (ME) is strongly attractive to males [14]. Based on the high potency and sex pheromone-like behavior of ME, trapping systems have been developed to manage *B. dorsalis* and as well as additional molecular mechanistic analysis of *B. dorsalis-*ME interactions [15, 16], but primarily with male flies. However, the oviposition by females is the main mechanism of fruit damage by insects. Therefore, it is important and necessary to develop new methods for trapping females and discovering oviposition stimulants may have greater potential in trapping gravid females [17].

Suitable oviposition sites are crucial for female insects due to their central role in successful development of offspring. During the oviposition, the olfactory cues from the external environment are important for female insects to make an appropriate choice for where to lay eggs. Previous studies showed that benzothiazole and 1-octen-3-ol could elicit oviposition behavior in gravid *B. dorsalis* and they are regarded as oviposition stimulants [17, 18]. The identification of oviposition stimulants would provide clues for understanding the co-evolution mechanism of *B. dorsalis* and their host plants, which could provide better methods for trapping female flies. However, the mechanism for how *B. dorsalis* percepts benzothiazole and 1-octen-3-ol still remains unknown. Previous research on oviposition stimulants were mainly focused on lepidopteran insects [17] and few studies have been reported in *B. dorsalis*. To better understand the mechanism of *B. dorsalis* perception of reported oviposition stimulants benzothiazole and 1-octen-3-ol, we sequenced and analyzed the antennal transcriptomes in both sexes of *B. dorsalis* with Illumina RNA sequencing (RNA-seq). We screened out and confirmed chemosensory DEGs, and female-biased chemosensory genes were selected to detect their expression profiles in virgin and mated female flies. In addition, we measured the expression levels of the female-biased chemosensory genes after exposed virgin and mated female flies to benzothiazole and 1-octen-3-ol for 2 h to determine chemosensory genes which may be responsible for oviposition stimulation. The evidence presented here provides fundamental data for male- and female-biased chemosensory genes and establishes an odorant exposure assay to lay a solid foundation for the further research into the molecular mechanisms of benzothiazole- and 1-octen-3-ol-mediated oviposition behavior in *B. dorsalis*.

## Results

### Transcriptome sequencing and assembly

A library of the male and female antenna was developed by Illumina sequencing with three replicates, which generated about 37.57 Gb of raw data. Each library was of good quality with Q20 and Q30 percentage of over 97.86 and 94.41%, respectively. After initial adaptor trimming and quality filtering, 3.72 × 10^8^ clean reads were obtained for sequencing from the six samples (Table 1). There were 8.67 × 10^5^ contigs with an N50 length of 425 bp assembled from clean data. The contigs were further assembled into 58,951 unigenes with a mean length of 774 bp using the paired-end joining and gap-filling method (Table 2). Among the total unigenes, the lengths of 48,337 unigenes (82%) were between 200 bp to 1000 bp, 10,614 unigenes (12%) were over 1000 bp, and no unigene was less than 200 bp.

**Table 1:** Statistical sequencing data of the RNA-Seq reads for the examined samples

Group Name	Female			Male		
	FAn1	FAn2	FAn3	MAn1	MAn2	MAn3
No. clean reads	5.99 × 10^7^	5.64 × 10^7^	6.04 × 10^7^	6.50 × 10^7^	6.97 × 10^7^	6.01 × 10^7^
No. clean bases	8.98 × 10^9^	8.46 × 10^9^	9.07 × 10^9^	9.74 × 10^9^	10.46 × 10^9^	9.01 × 10^9^
GC content (%)	39.05%	38.23%	38.16%	37.89%	38.49%	38.43%
Q20 (%)	97.93%	97.86%	97.87%	97.91%	97.96%	97.95%
Q30 (%)	94.48%	94.41%	94.41%	94.53%	94.61%	94.58%

*FAn* female antenna, *MAn* male antenna; 1, 2, 3 represent different biological replicates respectively. Clean reads, the filtered sequencing data; GC content, the percentage of the number of G and C in the total base; Q20 and Q30 refers to the error probability given to the identified base during the base calling process of the sequencing process

**Table 2:** Statistics of the assembled sequences

Group Name	Number
Total number of clean reads	3.72 × 10^8^
Total number of contigs	8.67 × 10^5^
Total assembled bases	5.57 × 10^10^
Total number of unigenes	58,951
Unigene N50 (bp)	1276
Maximum unigene length (bp)	19,935
Minimum unigene length (bp)	201
Average unigene length (bp)	774
Unigenes annotation in NR	31,045
Unigenes annotation in Swiss-Prot	20,777
Unigenes annotation in KEGG	7388
Unigenes annotation in GO	19,751
Unigenes annotation in KOG	16,103

### Annotation of predicted transcripts

Unigene sequences were annotated using BLASTX against the KOG, Swissprot, TrEML, GO, and non-redundant (NR) NCBI protein databases. All annotation details were obtained from the combined genes with similarities > 30%. In all, a total of 32,992 (55.97%) assembled unigene sequences showed annotation hits at least one of the protein or nucleotide databases and 5003 unigenes (8.5%) showed annotation hits in all databases. Among the annotations in these databases, the best hits were in the NR and TrEML databases where the number of annotated genes were 31,045 (52.66%) and 31,501 (53.44%), respectively (Table 2). According to the NR database, more than half of annotated unigenes (16,642 unigenes, 53.6%) showed homology with the oriental fruit fly, *B. dorsalis* (Figure S1), whereas there were 3782 unigenes (12.2%) matched to other species. A four-way Venn diagram plot (Figure S2) also showed the assembled unigenes annotated against NR, KEGG, Swissprot, and KOG databases. This diagram indicated that 6323 unigenes were annotated by all four databases, whereas 8806 unigenes had homologous sequences both in NR, Swissprot, and KOG databases.

### Analysis of DEGs

**Fig. 1:**
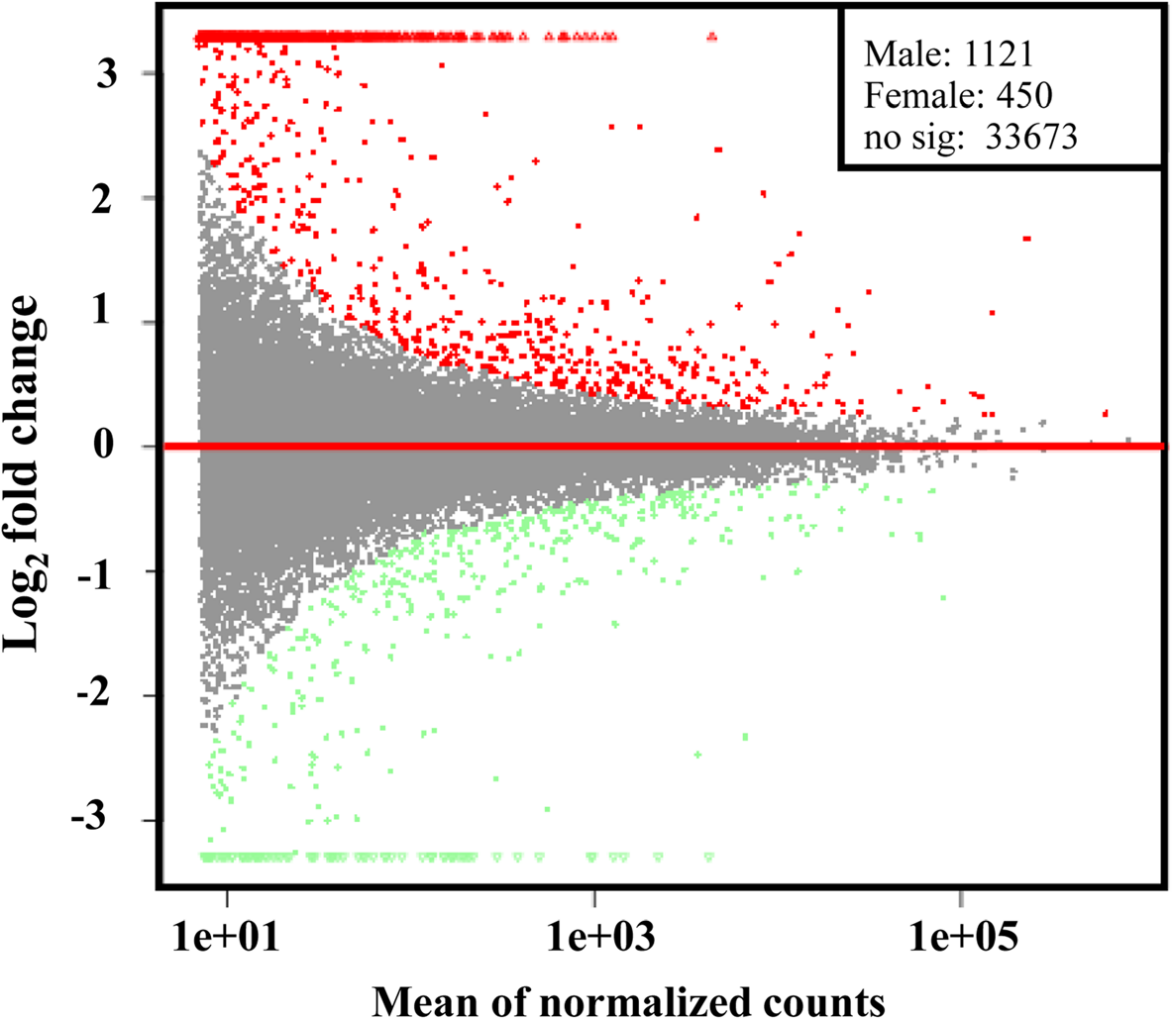
The DEG plot of FAn vs. MAn with *P*-value < 0.05. The X-axis indicates the mean of the normalized sequence count between the two groups, and the Y-axis represents the fold-change of gene expression levels (log_2_-fold change). The genes above the red line with red points are more highly expressed in the male adults and those below the red line with green points are more highly expressed in female adults. The grey points were the genes with no significant differences. The legend on the upper right shows the specific number of genes highly expressed in males and females. Abbreviations: FAn, female antenna; MAn, male antenna

DEG analysis was performed to gain the antennal transcriptome differences between the male and female adults. All of the DEGs were compared by normalizing the number of unambiguous tags in each library to RPKM (reads per kilobase mapped). To verify the reliability of the samples, the correlation between each sample was conducted by Pearson’s correlation analysis (Figure S3). The correlation coefficients range from 0.94 to 1, indicating the reliability of samples. Compared with the female group, the expression levels of 1571 unigenes were significantly different in the male group (Fig. 1). We identified 450 female-biased genes and 1121 male-biased genes from these DEGs. The fold change (log_2_ ratio) was calculated using the mean expression level of genes in male antenna divided to that in female antenna. Among DEGs, 24 genes were associated with olfaction including 10 OBPs, 12 ORs, 1 SNMP, and 1 GR. Their annotation information and fold changes are shown in Table 3.

**Table 3:** List of candidate olfactory genes from comparative antennal transcriptome

ID	FAn	MAn	log_2_ fold change	*P* adj	Gene description	Abbreviation
DN122377_c0_g1_i1	0.010	0.090	3.21	0.021	putative gustatory receptor 2a	GR 2a
DN154971_c8_g8_i2	424.03	278.82	−0.56	0.0023	sensory neuron membrane protein 1-like	SNMP 1
DN130398_c1_g1_i1	295.53	143.73	−1.00	2.72 × 10^−9^	odorant binding protein 3	OBP 3
DN120332_c0_g1_i1	1.04	6.21	2.68	1.33 × 10^−16^	odorant binding protein 3	OBP 5
DN135531_c41_g6_i1	3502.75	4547.85	0.42	0.00067	odorant binding protein 19d	OBP 19d
DN137330_c11_g9_i1	2987.73	3827.46	0.40	0.0015	odorant-binding protein 22	OBP 22
DN71860_c0_g1_i1	7.19	3.79	−0.86	0.046	odorant-binding protein 56d-like	OBP 56d-1
DN103452_c0_g1_i1	308.56	173.67	−0.77	4.78 × 10^−6^	odorant-binding protein 56d-like	OBP 56d-2
DN141472_c1_g1_i1	0.31	1.58	2.47	7.77 × 10^−9^	odorant-binding protein 57c	OBP 57c
DN117742_c0_g1_i1	0.36	4.33	3.60	2.69 × 10^−21^	odorant-binding protein 99a-like	OBP 99a
DN84733_c0_g1_i1	0.26	1.78	2.78	8.56 × 10^−5^	odorant binding protein c11	OBP c11
DN102208_c0_g1_i1	0.017	4.13	7.88	1.95 × 10^−15^	odorant binding protein c21	OBP c21
DN125498_c0_g1_i1	5.23	2.22	−1.19	0.0036	odorant receptor 7a-like	OR 7a-1
DN149599_c2_g17_i1	12.42	8.55	−0.52	0.0089	odorant receptor 7a-like	OR 7a-2
DN153156_c12_g20_i1	46.83	122.17	1.45	1.59 × 10^−9^	odorant receptor 7a-like	OR 7a-3
DN153156_c12_g2_i1	8.90	21.03	1.31	0.00041	odorant receptor 43b-like	OR 43b-1
DN136265_c1_g6_i2	34.73	2.02	−4.08	4.40 × 10^−19^	odorant receptor 43b-like	OR 43b-2
DN153156_c12_g35_i1	14.80	61.08	2.09	6.15 × 10^−15^	odorant receptor 43b-like	OR 43b-3
DN154906_c6_g4_i1	8.34	4.90	−0.72	0.041	odorant receptor 43b-like	OR 43b-4
DN77796_c0_g4_i1	4.93	1.16	−2.031	0.0012	orant receptor 43b-like	OR 43b-5
DN151752_c1_g26_i2	5.48	3.40	−0.67	0.015	odorant receptor 67d-like	OR 67d-1
DN151752_c1_g22_i1	38.69	22.02	−0.78	0.040	odorant receptor 67d-like	OR 67d-2
DN135169_c4_g1_i1	11.41	8.66	−0.38	0.033	odorant receptor 74a-like	OR 74a
DN125969_c0_g1_i1	0.014	0.16	3.61	0.0006	odorant receptor 94a-like	OR 94a

*ID* the gene ID, *FAn* female antenna, *MAn* male antenna. The values of FAn and MAn are the average expression levels of three biological replications; the fold change value was obtained by calculating the shrinkage model in the differential analysis software (DESeq2) and fold change represents the fold change of expression level from females to males; *P* adj, the corrected *P*-value; Gene description, the description of gene function

### qRT-PCR validation

**Fig. 2:**
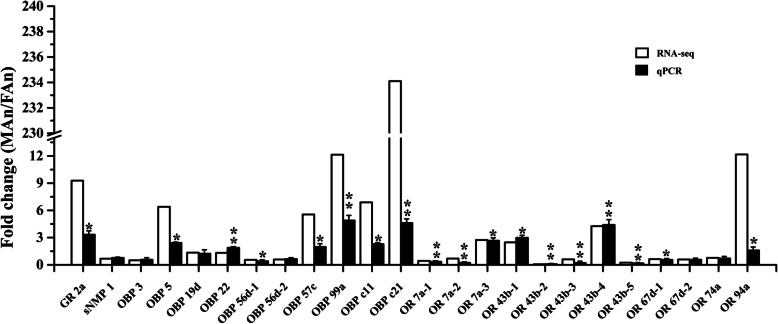
Validation of candidate olfactory genes by qRT-PCR. The expression of the fold-change measured by RNA-seq (*n* = 3) and qRT-PCR (*n* = 4) is indicated in white and black columns, respectively. Values shown in black columns indicate mean ± SE from four independent experiments. The asterisks above the standard error bars indicate statistically significant differences among the expression level of males and females in the qRT-PCR (*P < 0.05; **P < 0.01) (independent-samples *t*-test)

To confirm the DEG results from Illumina sequencing, we confirmed the 24 chemosensory genes by qRT-PCR (Fig. 2). Among them, 12 genes were highly expressed in the female antennae and the other 12 genes were highly expressed in the male antennae. As expected, the results of the qRT-PCR were basically consistent with the RNA-Seq data. All candidate chemosensory genes had similar expression change trends as the DEG analysis from Illumina sequencing. However, there were some genes whose fold changes were different between DEG analysis and qPCR results. For instance, a 9.28-fold change in DEG analysis versus about 3.3-fold in the result of qPCR for GR 2a, a 6-fold change in DEG analysis versus about 2.4-fold in qPCR detection for OBP 5, and 12.14-fold change in DEG analysis versus about 2-fold in the result of qPCR for OBP 99a. The largest difference in fold change between DEG analysis and qPCR validation was OBP c21, which showed 234-fold change in DEG analysis and 4.6-fold in qPCR validation. Meanwhile, the 24 selected genes showed a significant correlation (*R*^*2*^ = 0.90, *P* < 0.01, Spearman correlation coefficient) between RNA-seq and qPCR results (Figure S4). It indicated the reliable of RNA-seq data in this study.

**Fig. 3:**
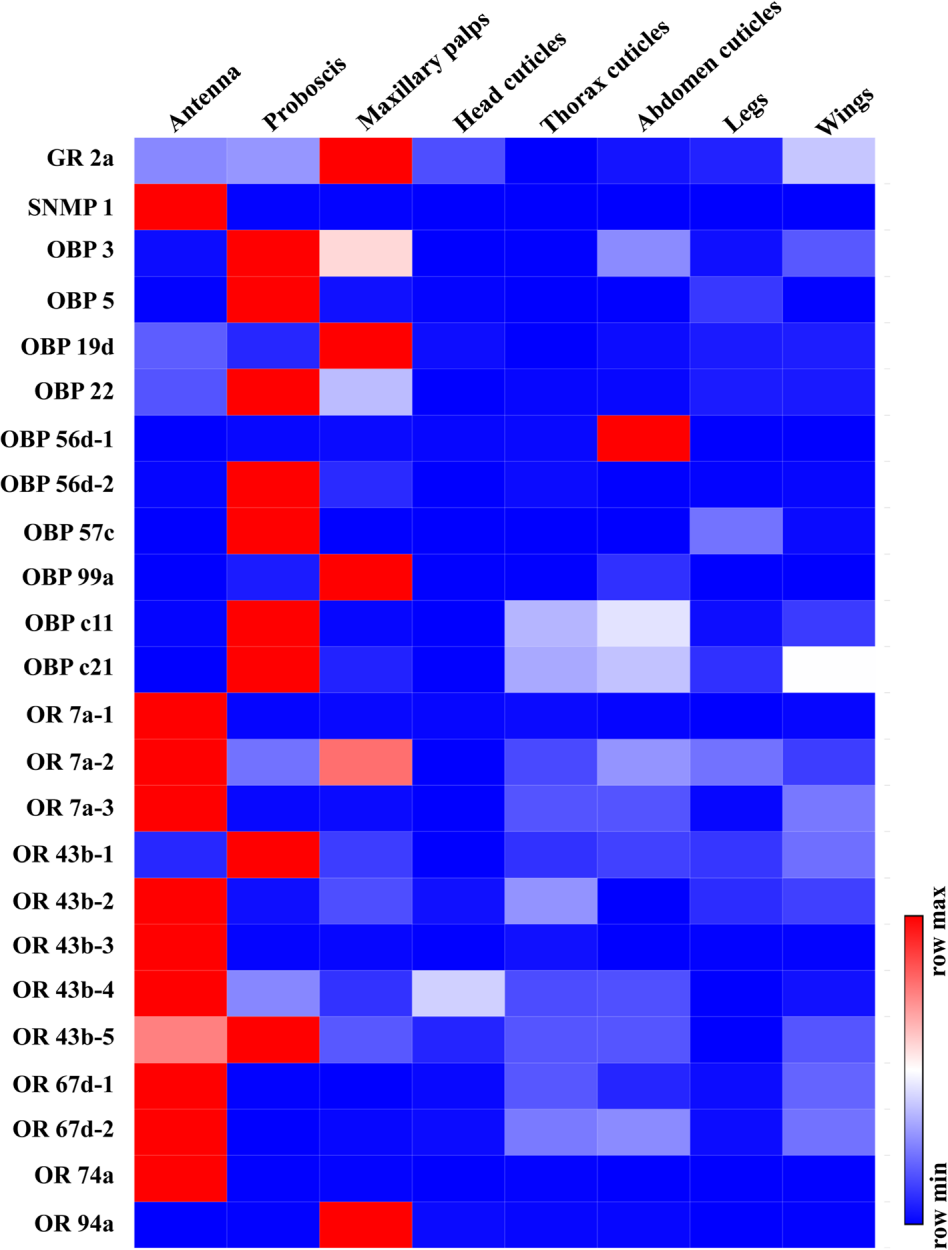
The expression patterns of candidate olfactory genes in different body segments. Body segments from female flies included eight parts: antenna, proboscis, maxillary palps, head cuticles, thorax cuticles, abdomen cuticles, legs, and wings. The heatmap was generated using the online website https://software.broadinstitute.org/morpheus/. Each row represents a gene, and each column represents a tissue. A relative color scheme used the minimum and maximum values in each row to convert values to the intensity of colors. Red represents the relative higher expression level, and blue is the relative lower expression level

In addition, the expression patterns of these 24 candidate genes were further investigated in different body parts of *B. dorsalis* across eight kinds of tissue (Fig. 3). As indicated by the qRT-PCR, all candidate genes were highly expressed in tissues that are rich in sensilla like antenna, proboscis, and maxillary palps, except for OBP 56d-1. OBP 56d-1 expression was highest in abdomen cuticles.

**Fig. 4:**
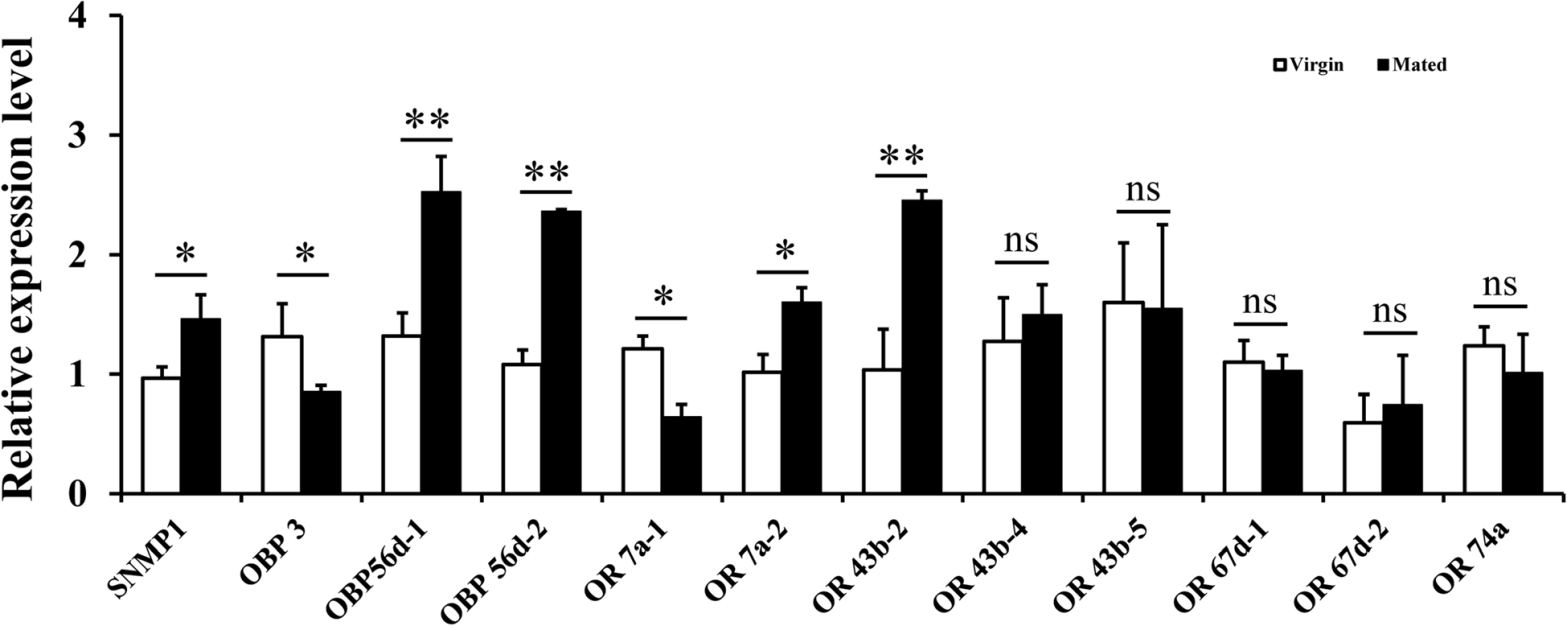
The expression profile of 12 female-biased chemosensory genes in virgin and mated flies. The white and black columns represent the virgin and mated flies, respectively. Values shown in figure represent mean ± SE from four independent experiments. The asterisks indicate statistically significant differences among the virgin and mated flies in the qRT-PCR (*P < 0.05; **P < 0.01) (independent-samples *t*-test)

To investigate the expression profile of the 12 female-biased genes in virgin and mated flies, the qPCR detection was performed with 15-day-old virgin and mated flies, respectively (Fig. 4). The expression level of five candidate genes was significantly upregulated after mating including SNMP 1, OBP 56d-1, OBP 56d-2, OR 7a-2, and OR 43b-2. In contrast, the expression levels of OBP 3 and OR 7a-1 decreased significantly after mating. Expression levels of the other five candidate genes showed no significant differences between virgin and mated flies.

### Transcript level detection of female-biased DEGs after odorant exposure

**Fig. 5:**
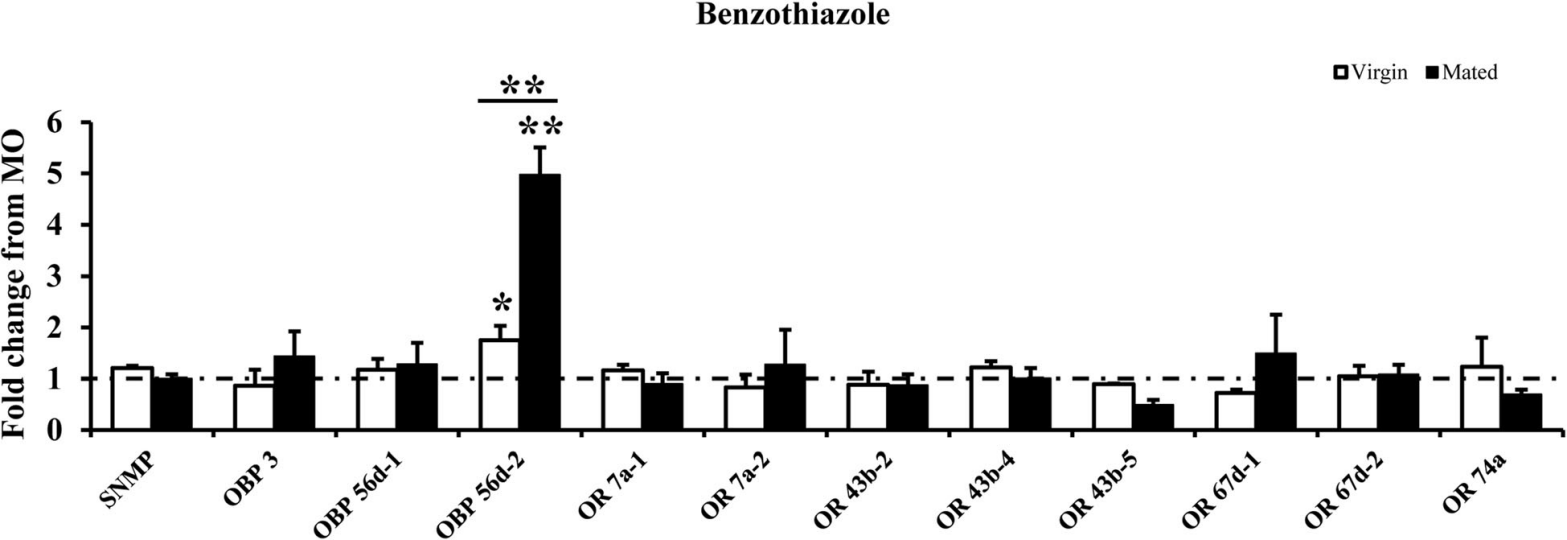
Effect of exposure to benzothiazole on transcript levels of 12 female-biased chemosensory genes. The fold change of expression level from benzothiazole treatment to MO treatment in virgin and mated flies is indicated in white and black columns, respectively. Values shown in figure represent mean ± SE from three independent experiments. The asterisks above the standard error bars indicate statistically significant differences among the expression level of benzothiazole treatment to MO treatment, the asterisks between white and black columns indicate statistically significant differences among virgin and mated groups (*P < 0.05; **P < 0.01) (independent-samples *t*-test)

**Fig. 6:**
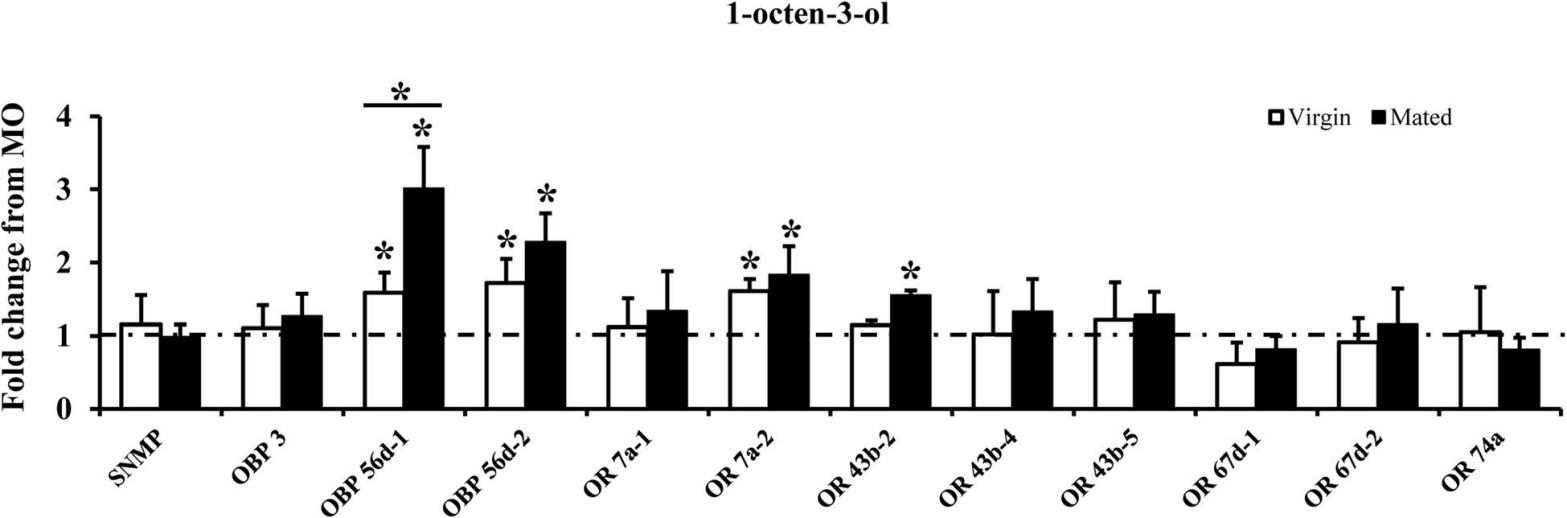
Effect of exposure to 1-octen-3-ol on transcript levels of 12 female-biased chemosensory genes. The fold change of expression level from 1-octen-3-ol treatment to MO treatment in virgin and mated flies is indicated in white and black columns, respectively. Values shown in figure represent mean ± SE from three independent experiments. The asterisks above the standard error bars indicate statistically significant differences among the expression levels of 1-octen-3-ol treatment to MO treatment, the asterisks between white and black columns indicate statistically significant differences among virgin and mated group (*P < 0.05; **P < 0.01) (independent-samples *t*-test)

To confirm the characteristic of oviposition stimulants benzothiazole and 1-octen-3-ol for female flies, the transcript levels of the screened 12 female-biased genes were measured after exposing virgin and mated flies to benzothiazole and 1-octen-3-ol for 2 h. The results showed that after exposure to benzothiazole for 2 h, the expression level of OBP 56d-2 increased significantly in virgin flies and was about 1.58-fold changed from MO exposure (Fig. 5). In mated flies, the expression level of OBP 56d-2 increased significantly as well after exposure to benzothiazole (Fig. 5). However, the fold change was much higher, about a 5-fold change. As for 1-octen-3-ol exposure, the expression level of OBP 56d-1, OBP 56d-2, and OR 7a-2 increased in virgin flies (Fig. 6). For mated flies, the expression level of these three genes increased as well, and the fold changes were much higher (Fig. 6). In addition, except for the three changed genes in virgin female flies, the expression level of OR 43b-2 increased when exposed mated females to 1-octen-3-ol. However, the fold change was lower the other three genes.

## Discussion

In this study, the sequencing quality was reliable according to our evaluation by the Illumina’s base-caller Bustard software. More than 94.4% of clean reads had quality scores higher than the Q30 level (an error probability of 0.1%) (Table 1). Compared with our previous study [19], we have substantially expanded our dataset of chemosensory DEGs in different sexes of *B. dorsalis* antenna.

Sexual dimorphisms in olfaction-mediated behaviors have drawn extensive attention both in terms of understanding insect behavior and as potential mechanisms for insect pest control. However, it remains still unknown whether males and females have the same ability of chemoperception. DEG analysis generated from the comparative transcriptomics was proven to be a powerful tool to explore sex-biased genes involved in the sexual dimorphisms of insect behaviors [9, 20, 21]. Insects perceive the surrounding through different organs [22], among which antennae are the most important and play a central role in the life of the insect. Therefore, antennae were used to conduct comparative transcriptomic analysis between both sexes in this study. In the present study, we screened out 24 chemosensory DEGs, it was similar to a previous study which identified 27 sex-biased chemosensory genes in *Drosophila suzukii* antennae [23].

All of the candidate chemosensory genes detected by qRT-PCR showed similar change trends as indicated by DEG analysis, suggesting that a DEG approach can be a powerful way to screen out candidate genes in a high-throughput manner. However, there were some fold-change magnitude differences between qRT-PCR and DEG analysis. The reason may be due to sensitivity bias between two methods or different statistical approaches and thresholds in qRT-PCR and DEG analysis [24]. In addition, we also examined the expression profiles of these chemosensory genes in different appendages in female adults. As indicated by the results, with exception to OBP 56d-1, all candidate genes were high expressed in tissues rich in sensilla like antenna, proboscis, and maxillary palps, suggesting these genes may play an important role in olfaction or gustation. However, the expression of OBP 56d-1 was highest in abdomen cuticles. Therefore, we inferred that abdomen cuticles may contain many sensilla, especially on the ovipositor. A recent study showed that *HassOR31* highly expressed in ovipositor was involved in detecting host-plant volatiles for determining precise oviposition sites [25]. This gene may exert a function associated with host-plant volatile detection or stimulation of egg lay.

Olfactory cues influence insects in many different ways [26]. For instance, oviposition is one of the most important female behaviors and has great influence on the fitness of insect species. Since egg distribution is determined by adult females, searching for suitable oviposition sites plays an important role in oviposition behaviors for gravid females [27]. In addition, oviposition stimulants could enhance fecundity of the gravid females [18]. Thus, whether these sexually dimorphic behaviors are associated with the sex-biased expression of chemosensory genes needs to be clarified. Previous studies indicated that several pheromone receptors, which are specifically expressed in male antenna, are responsible for the male attraction by sex pheromones produced by females in *Heliothis virescens* [28]. In *Maruca vitrata*, *MvitGOBP1–2* showed the highest expression levels in female antennae, and it was reported to bind with some floral volatiles, which may effectively attract female moths [29]. In addition, dsRNA mediated knock down of the male-biased genes *OBP 99a* dramatically reduced courtship behavior in *B. dorsalis* males [30]. Based on the previous studies, we speculated that the chemosensory genes responsible for special odorants, which could elicit sexual behaviors, may have sex-biased expression. Therefore, the differentially expressed chemosensory genes presented in this study should be involved in some sexual behaviors.

It has been reported that benzothiazole and 1-octen-3-ol serve as oviposition stimulants in *B. dorsalis* and may attract the gravid females to lay more eggs [17, 18]. Therefore, we screened the female-biased chemosensory genes and compared the expression of these genes in the virgin and mated flies. We found that the expression of five candidate genes (SNMP 1, OBP 56d-1, OBP 56d-2, OR 7a-2, and OR 43b-2) were upregulated after mating. The results indicated that these five genes may be involved in perception of volatiles from host plants for seeking oviposition site. Similar results have been observed in a study of *Dendrolimus punctatus* [31]. The previous study showed that chemosensory genes could be activated by a given odorant in vivo and that odorants could induce a fast change in the transcription level of activated chemosensory genes [32, 33]. In this study, odorant exposure assays showed that OBP 56d-2 was upregulated when the flies were exposed to benzothiazole. Furthermore, the fold-change of expression level in mated females was much higher than that in virgin females. Similar results were observed for three chemosensory genes (OBP 56d-1, OBP 56d-2, and OR 7a-1) in the 1-octen-3-ol exposure assay. This suggested that the mated flies were more sensitive to oviposition stimulants, which is entirely consistent with their biological characteristics. In addition, all these changed genes have higher expression levels after mating. Mated flies would rapid change their physiology and behavior, the gravid state influences their perception of odors and taste along with the increased feeding and egg laying behaviors [34, 35]. In addition, a previous study showed that 1-octen-3-ol and benzothiazole are more attractive to *B. dorsalis* after mating, and EAG responses are also stronger after mating [36]. Taken together, we speculated that OBP 56d-2 might be responsible for perception of benzothiazole, whereas OBP 56d-1, OBP 56d-2, OR 7a-1, and OR 43b-2 may be responsible for 1-octen-3-ol perception in *B. dorsalis*, which will facilitate the location of oviposition sites in this fly. Nevertheless, more solid evidence should be further pursued to confirm the relationship between these candidate chemosensory genes and the oviposition stimulants.

## Conclusion

This study sequenced the antennal comparative transcriptome of *B. dorsalis* in different sexes and constructed DEG libraries. From the transcriptomic analysis, we screened 1151 DEGs, including 24 chemosensory genes. Among these differentially expressed chemosensory genes, there were 12 female-biased genes and five of them were upregulated after mating. The expression level of OBP 56d-2 increased after exposed to benzothiazole for 2 h, and the expression level of OBP 56d-1, OBP 56d-2, OR 7a-1, and OR 43b-2 was upregulated after exposed to 1-octen-3-ol for 2 h. The fold-change of expression level was higher in mated flies, indicating mated flies might be more sensitive to oviposition stimulants. In addition, OBP 56d-1, OBP 56d-2, OR 7a-1, and OR 43b-2 might be responsible for percepting oviposition stimulants and may be involved in locating oviposition sites. The results from this study have laid a solid foundation for the further research regarding the mechanism of benzothiazole-and 1-octen-3-ol-mediated oviposition behavior in *B. dorsalis.*

## Methods

### Insects

Oriental fruit flies were reared at the Key Laboratory of Entomology and Pest Control Engineering in Chongqing, China. The laboratory colony of* B. dorsalis* was originally collected from Haikou, Hainan province, China, in 2008. The insects were reared in the laboratory at 27 ± 1 °C, 70 ± 5% relative humidity, with a 14 h light: 10 h dark photoperiod [37]. Adults were fed an aqueous artificial diet of yeast powder, honey, sugar, ascorbic acid [38].

### RNA isolation, cDNA library preparation, and Illumina sequencing

Samples were collected from five-day-old adult *B. dorsalis* and divided into two groups by sex. Each group had three independently biological replications and each replication contained antennae from 100 individuals. The antennae were dissected on the chilled ice and immediately frozen in liquid nitrogen.

Total RNA was extracted from the antennae of *B. dorsalis* using TRIzol reagent (Invitrogen Life Technologies, Carlsbad, CA) with DNase (Promega, Madison, WI) treatment to remove genomic DNA, followed by a phenol-chloroform extraction [39]. The purity of all RNA samples was assessed by absorbance ratios of OD_260/280_ and OD_260/230_, whereas the integrity of RNA was verified by electrophoresis on a 1.0% agarose gel and the concentration of RNA was determined by Nanodrop One (Thermo Fisher Scientific, Madison, WI). The cDNA library construction for Illumina sequencing was conducted with 4 μg RNA from each sample by using the Illumina TruSeq RNA Sample Preparation Kit by the manufacturer instructions. Sample sequencing was conducted on Illumina HiseqTM 2000 using paired-end technology with the PE150 sequencing strategy.

### De novo assembly and bioinformatics analysis

The raw reads in the FASTQ format were cleaned by removing adapter sequences [40]. Short or low-quality reads containing > 5% unknown nt “N” and reads with 20% quality value < 10 were removed from datasets to acquire more reliable results. The clean reads were de novo assembled into contiguous sequences (contigs) with the Trinity Method [41] to recover more full-length transcripts across a broad range of expression levels and sequencing depths with an optimized k-mer length of 25 for de novo assembly [41]. The assembled contigs were further clustered using TGI Clustering Tool [42] and the transcripts were clustered based on nucleotide sequence identity.

### The annotation and analysis of DEGs

All raw sequence reads were filtered using the Illumina pipeline before mapping reads to the reference transcriptome database. Low-quality reads were removed from the data analysis. Low-quality reads include those which had more than 10% ambiguous bases and the quality value of those in more than 50% reads were ≤ 5. The level of gene expression was determined by normalizing the number of unambiguous tags in each sample to RPKM [42]. The relative gene expression in male antenna or female antenna was equal to the mean of three biological samples from each group. The fold change was calculated as fold change with log_2_ ratio. The differentially expressed transcripts between samples were identified using a Bayesian algorithm [43]. The false discovery rate (FDR) method was used to determine the threshold of the *P*-value in multiple tests and analyses. Genes with an adjusted *P*-value < 0.05 were for comparisions of gene expression and genes expressed at different levels across the samples were further annotated by GO function.

### Quantitative real-time PCR validation of the DEGs

To verify the DEG results, the differentially expressed chemosensory genes were analyzed by qRT-PCR assay. Gene-specific primers and the target genes are listed into Table S1. The male and female antenna of five-day-old adult *B. dorsalis* were collected and each group contained 50 antennae with four biological replicates. RNA was extracted as described above. Total RNA (1 μg) was treated with RNase-free DNase (Promega, Madison, WI, USA) to remove the genomic DNA at 37 °C for 30 min. First strand cDNA was synthesized with the PrimerScript RT Reagent kit (Takara, Dalian, China) following the manufacturer instructions.

The qRT-PCR was performed using CFX384TM Real-Time System (Bio-Rad, Singapore, Jurong) with a 10 μL reaction volume. The amplification efficiency of each primer was first determined with a standard curve based on a 5-fold cDNA dilution series. Then, the expression profile in male and female antennae was performed using the appropriate primers. Each 10 μL reaction mixture contain 5 μL SYBR supermix (Novoprotein, Shanghai, China), 3.9 μL nuclease-free water, 0.5 μL of each cDNA sample (approximately 300 ng/μL), and 0.3 μL forward and reverse primers (10 μM). The procedure for PCR was as follows: an initial denaturation at 95 °C for 2 min, followed by 40 cycles at 95 °C for 15 s, and incubation at 60 °C for 30 s. A melt curve was performed to ensure the specificity from 60 to 95 °C [39]. The double internal reference genes α-tubulin (GenBank: GU269902) and ribosomal protein S3 (rps 3, GenBank: XM_011212815) were applied because of their stable expression level in *B. dorsalis*. The relative expression was calculated with Biogazelle qBase software and the data were analyzed with SPSS 16.0 using one-way analysis of variance (ANOVA) followed by Turkey’s honestly significantly difference test (*P* < 0.05).

### Tissues and odorant-induced expression profiling

Different tissues including antenna, proboscis, maxillary palps, head cuticles, thorax cuticles, abdomen cuticles, legs, and wings were dissected from female flies. In addition, the head of 15-day-old virgin and mated female flies were dissected separately. The total RNA of these tissues was extracted for four biological replicates using the method as mentioned above. To detect the expression profile of the female-biased genes after the benzothiazole and 1-octen-3-ol exposure, a bioassay was conducted using 5% (vol/vol) benzothiazole (≥96%, sigma, USA) and 1-octen-3-ol (≥98%, sigma, USA). The odorants were diluted in mineral oil (MO, sigma, USA) whereas the MO-only served as the control. Odorants were applied to 15-day-old virgin and mated female flies. The assay was performed at a 700 mL transparent cage in a 27 °C incubator. Filter strips (5 × 1 cm) loaded with 50 μL 5% benzothiazole, 1-octen-3-ol, or MO were placed at the center of the cage. Each cage contained 50 female flies. Before odorant exposure, the flies were starved for 8 h. Exposure started in the morning at 9 am and lasted for 2 h. The exposure of different odorants was conducted in different incubators with three replicates. After the exposure, antennae of flies were dissected on ice and prepared for tissue extraction. The total RNA and first strand cDNA were obtained as described above. The tissues and odorant-induced expression profilings were detected by qPCR.

## Supplementary Information

**Additional file 1: Figure S1.** Homology analysis of unigenes in *B. dorsalis* for species distribution. The species distribution was shown as percentage of total homologous sequences in the NCBI NR protein database. The different colors represented different species.

**Additional file 2: Figure S2.** Venn diagram for four databases: NR, KEGG, Swissport, and KOG.

**Additional file 3: Figure S3.** Heat map of correlation between samples. A relative color scheme used to represent the correlation coefficient between samples. MAn represents male antenna, FAn represents female antenna.

**Additional file 4: Figure S4.** Pearson’s correlation of gene expression fold changes from female flies (Log_2_ fold change) measured using RNA sequencing (RNA-seq) and real-time quantitative PCR (qPCR). MAn represents male antenna, FAn represents female antenna.

**Additional file 5: Table S1.** Primer sequences of candidate olfactory genes used for qRT-PCR.

## Data Availability

All datasets for this study are included in the manuscript and/or the supplementary files. All row reads of the transcriptome had been archived at the Short Read Archive (SRA) in NCBI under project accession number PRJNA657376 and biosample accession numbers SRR12473599-SRR12473604.

## Abbreviations

RNA-seq: RNA sequencing
DEGs: Differentially expressed genes
OSNs: Olfactory sensory neurons
OBPs: Odorant binding proteins
SNMP: Sensory neuron membrane proteins
CSPs: Chemosensory proteins
ODEs: Odorant degrading enzymes
ORs: Odorant receptors
GRs: Gustatory receptors
IRs: Ionotropic receptors
ME: Methyl eugenol
RPKM: Reads per kilobase mapped
FDR: False discovery rate
MO: Mineral oil
