# Supplementary figures and images for: Comparative transcriptomic analysis reveals female-biased olfactory genes potentially involved in plant volatile-mediated oviposition behavior of Bactrocera dorsalis
Source: BMC Genomics. 2021 Jan 6;22:25. doi: 10.1186/s12864-020-07325-z (PMC7789660; doi:10.1186/s12864-020-07325-z)

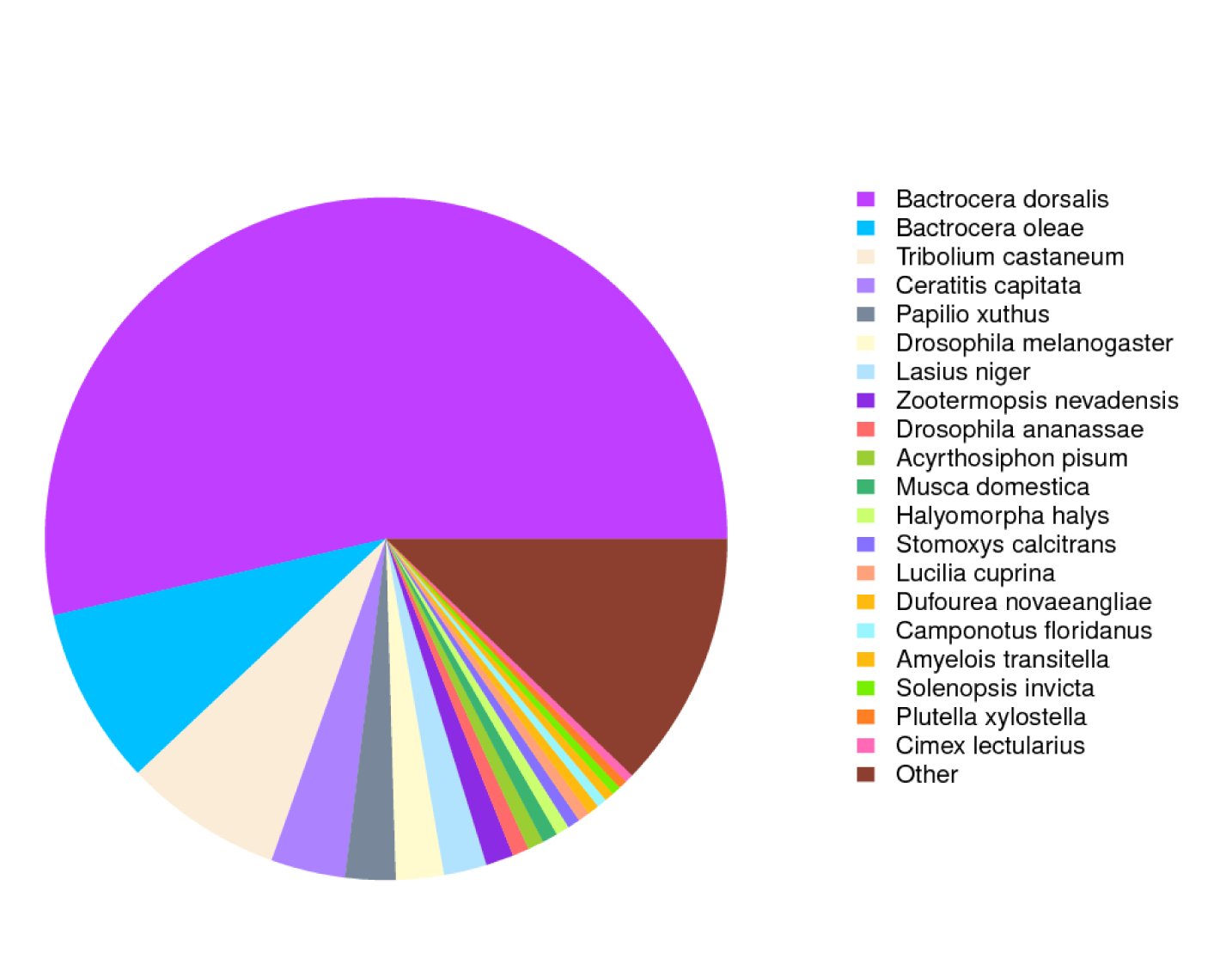

Supplement: Supplementary file 1 — Additional file 1: Figure S1. Homology analysis of unigenes in B. dorsalis for species distribution. The species distribution was shown as percentage of total homologous sequences in the NCBI NR protein database. The different colors represented different species. [file 12864_2020_7325_MOESM1_ESM.tif]

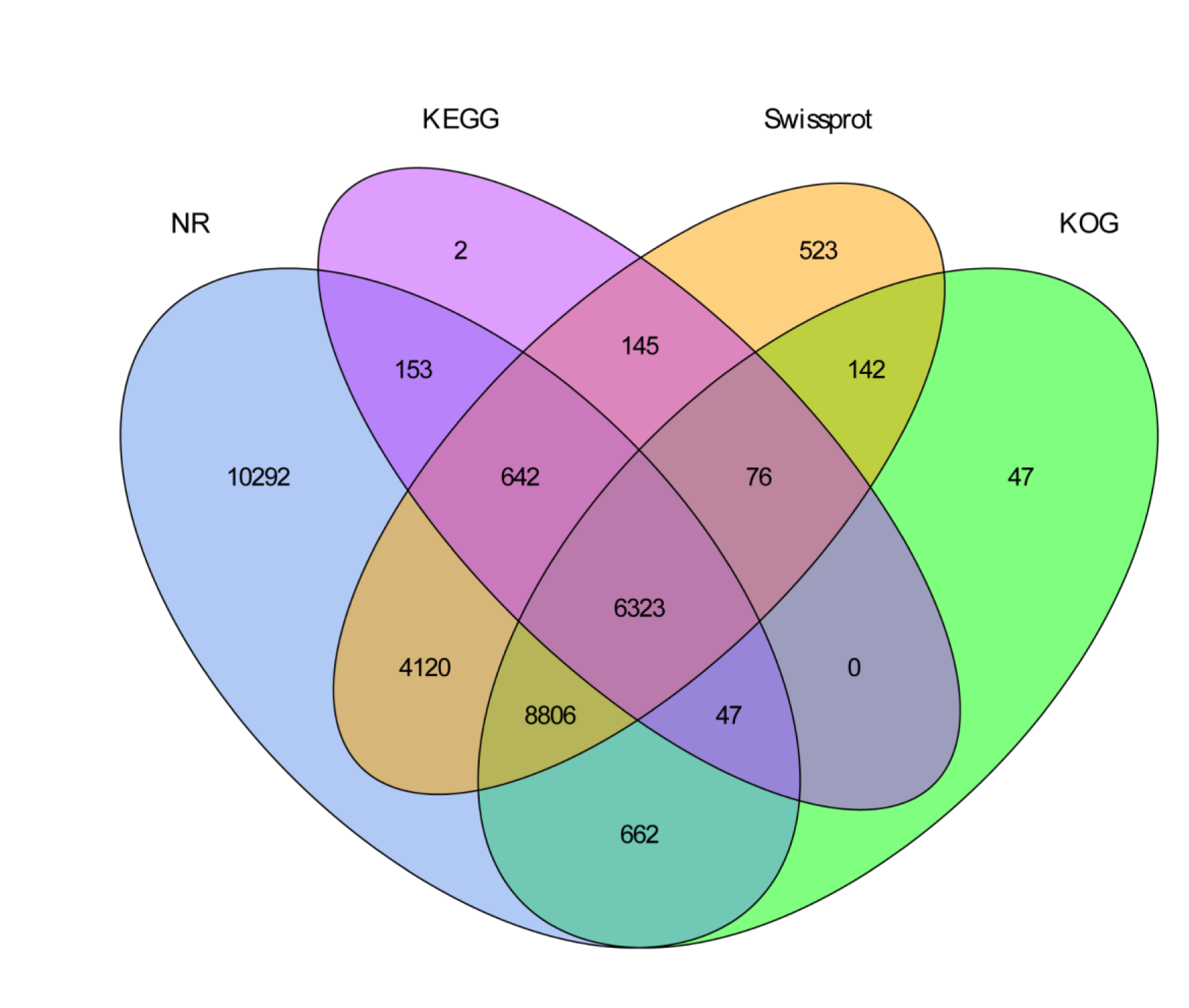

Supplement: Supplementary file 2 — Additional file 2: Figure S2. Venn diagram for four databases: NR, KEGG, Swissport, and KOG. [file 12864_2020_7325_MOESM2_ESM.tif]

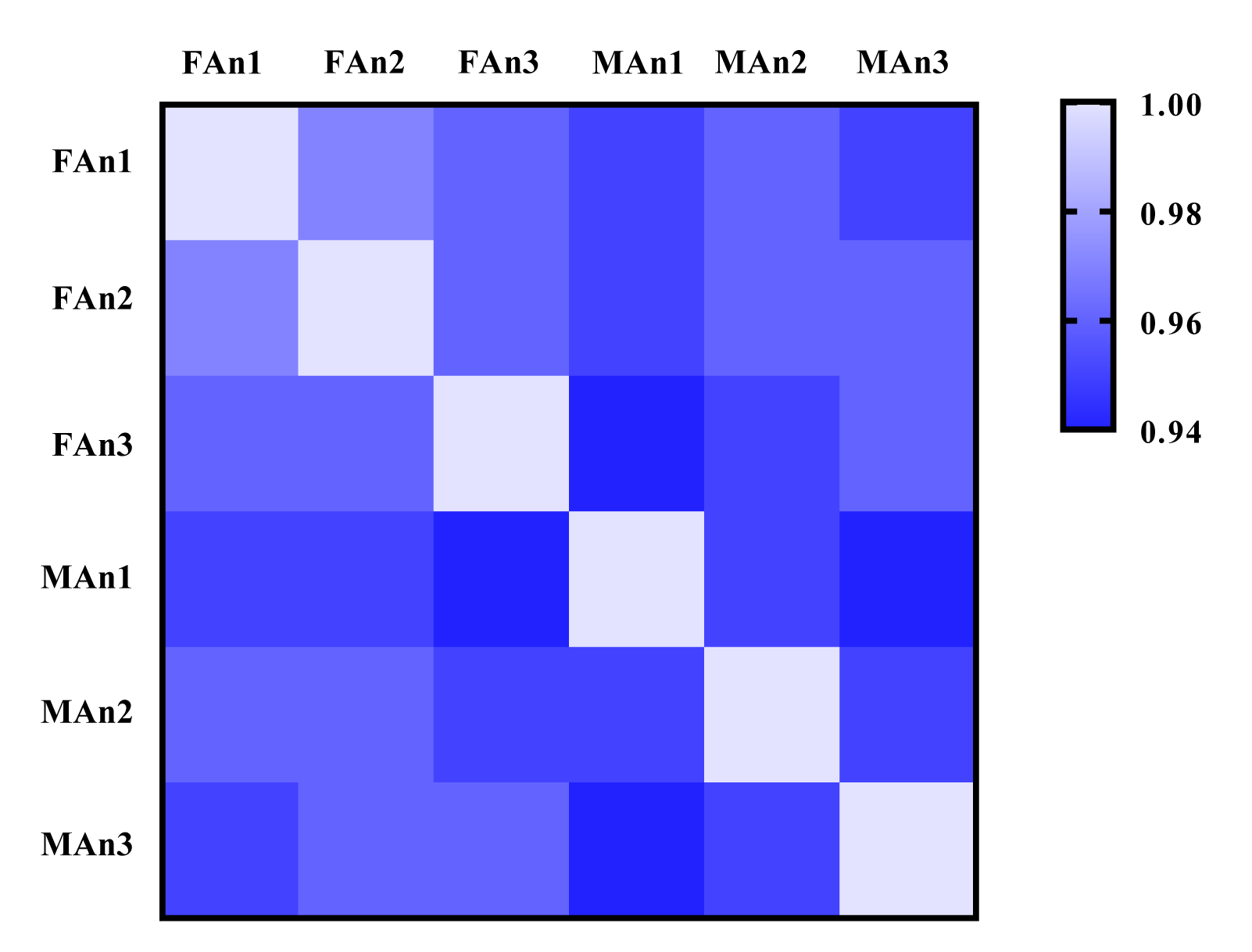

Supplement: Supplementary file 3 — Additional file 3: Figure S3. Heat map of correlation between samples. A relative color scheme used to represent the correlation coefficient between samples. MAn represents male antenna, FAn represents female antenna. [file 12864_2020_7325_MOESM3_ESM.tif]

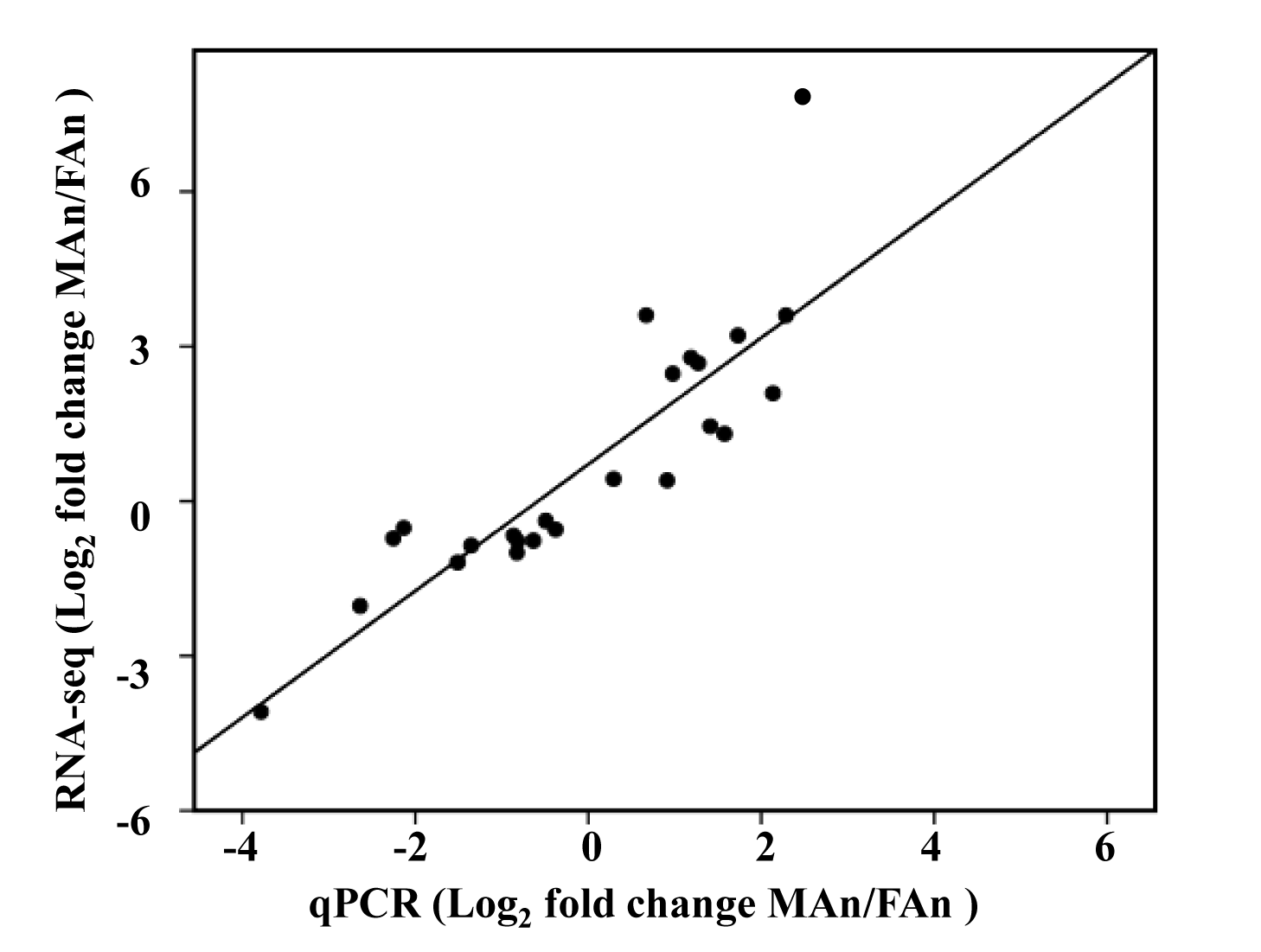

Supplement: Supplementary file 4 — Additional file 4: Figure S4. Pearson’s correlation of gene expression fold changes from female flies (Log2 fold change) measured using RNA sequencing (RNA-seq) and real-time quantitative PCR (qPCR). MAn represents male antenna, FAn represents female antenna. [file 12864_2020_7325_MOESM4_ESM.tif]
