# Supplementary material for: Comparative transcriptomic analysis reveals female-biased olfactory genes potentially involved in plant volatile-mediated oviposition behavior of Bactrocera dorsalis
Source: BMC Genomics. 2021 Jan 6;22:25. doi: 10.1186/s12864-020-07325-z (PMC7789660; doi:10.1186/s12864-020-07325-z)
Supplement: Supplementary file 5 — Additional file 5: Table S1. Primer sequences of candidate olfactory genes used for qRT-PCR. [file 12864_2020_7325_MOESM5_ESM.docx]

**Table S1. Primer sequences of candidate olfactory genes used for real-time qPCR**

| Gene name | Primer name | Primer-sequence (5′–3′) | Amplification  Efficiency |
| --- | --- | --- | --- |
| GR 2a | qGR 2a -F | CATCTGCTCTTTCTCACCGC | 98.7 |
|  | qGR 2a -R | GTTTGGGGCCTCATTCGAAG |  |
| SNMP 1 | qSNMP 1-F | GGAGAAATTTCCCATCGCACT | 97.3 |
|  | qSNMP 1-R | GCGTCCTCATCCTCAAAGTC |  |
| OBP 3 | qOBP 3-F | TGCTTTAAGGAAACCGGTGC | 101.8 |
|  | qOBP 3-R | TTAGCTTTCTCCTCGCCGAT |  |
| OBP 5 | qOBP 5-F | TTTTGAGTTACAGCCCACGC | 95.6 |
|  | qOBP 5-R | CGGGCAACTTCAAACCTGAT |  |
| OBP 19d | qOBP 19d-F | ACCGGAGAGCTGCTTTATGT | 97.9 |
|  | qOBP 19d-R | TCGCTGAGGACTGTAAAGGG |  |
| OBP 22 | qOBP 22-F | GGTGCGATGAATGGTGATGG | 105.4 |
|  | qOBP 22-R | GCCCCACTACAAAGCCAATC |  |
| OBP 56d-1 | qOBP 56d-1-F | GCCCAATTTACCTAGCATGCA | 104.3 |
|  | qOBP 56d-1-R | GTTGACCCCGGAGCAGATAC |  |
| OBP 56d-2 | qOBP 56d-2-F | GTGTCACATTCGTCGGTACC | 96.3 |
|  | qOBP 56d-2-R | GCTTGAAAGAAACCGGTGCT |  |
| OBP 57c | qOBP 57c-F | GCTTGAAAGAAACCGGTGCT | 99.7 |
|  | qOBP 57c-R | GAGGGATTGCATGAATGGGG |  |
| OBP 99a | qOBP 99a-F | GCTGAAGATGAATGGAGACCG | 93.9 |
|  | qOBP 99a-R | CAAAGCAAAACCTTACGCGC |  |
| OBP c11 | qOBP c11-F | TGGCTGAGGACACCGATAAG | 98.9 |
|  | qOBP c11-R | CCTGGCCCTAATCTTGTCCA |  |
| OBP c21 | qOBP c21-F | CCGACACTCACTGCTCTTGA | 100.5 |
|  | qOBP c21-R | TTCTCAACGGTGCACATCAT |  |
| OR 7a-1 | qOR 7a-1-F | TCAGGAATTGTTGGAGGCGA | 98.1 |
|  | qOR 7a-1-R | GCAACTTGGCACTGTCAACT |  |
| OR 7a-2 | qOR 7a-2-F | CACGATGCGAAGACAGTGAG | 95.2 |
|  | qOR 7a-2-R | CGGTGACTGCCTCATATTGC |  |
| OR 7a-3 | qOR 7a-3-F | GCGTTTGTCCATATCCATCC | 91.7 |
|  | qOR 7a-3-R | CCGATACGATGCACAAGGTT |  |
| OR 43b-1 | qOR 43b-1-F | GTGCCTAAACGACTGATGCG | 93.2 |
|  | qOR 43b-1-R | CATCAGCCCCTACGACACA |  |
| OR 43b-2 | qOR 43b-2-F | CGTTCTCGTCGTCTGTTAATCT | 97.4 |
|  | qOR 43b-2-R | GTCCACTCTCATACCACGACT |  |
| OR 43b-3 | qOR 43b-3-F | ACAGTGTCGTAGGGGCTGAT | 101.5 |
|  | qOR 43b-3-R | GCGAAAAGACTTGCAGAAGC |  |
| OR 43b-4 | qOR 43b-4-F | ATTTGTATGGCAGCACCACA | 94.1 |
|  | qOR 43b-4-R | TGCATCCATTGAGGCTTGTA |  |
| OR 43b-5 | qOR 43b-5-F | GTGTCCGTTCACTTGCATTG | 98.3 |
|  | qOR 43b-5-R | ATTCTGATAGCGTCCGTCCA |  |
| OR 67d-1 | qOR 67d-1-F | GGCCTATACGCTGGTGATGT | 96.4 |
|  | qOR 67d-1-R | CGCCGTGGTGCATATAATGT |  |
| OR 67d-2 | qOR 67d-2-F | AACCGCTTTTCTTTCCACAA | 95.6 |
|  | qOR 67d-2-R | CGCCTGATGCTAACACAATC |  |
| OR 74a | qOR 74a-F | GGCGTCCAAAGATCAGCAAT | 97.8 |
|  | qOR 74a-R | TCGCCAAATCAATGCAGGAG |  |
| OR 94a | qOR 94a-F | TGGCCATACACTCAACTCGT | 94.9 |
|  | qOR 94a-R | AATGCGTGGCCTCTATTCCT |  |
